# Discursive structures of global development governance: a mixed-methods network analysis of an academic deliberative space in the context of FfD4

**DOI:** 10.3389/fsoc.2026.1813491

**Published:** 2026-05-29

**Authors:** Jesús Delgado-Baena, Juan de Dios García-Serrano, Tomás Diestre-Mejías, Pablo Iglesias-Nicasio

**Affiliations:** 1 Department of Social Work and Social Services, Faculty of Social Sciences, Universidad Pablo de Olavide, Seville, Spain; 2 Department of Economics, Quantitative Methods and Economic History, Universidad Pablo de Olavide, Seville, Spain; 3 Doctoral Program in Social Sciences, Universidad Pablo de Olavide, Seville, Spain

**Keywords:** critical development theory, development discourse, development financing, discourse network analysis, ethical governance, global governance, social network analysis

## Abstract

This article examines development financing as a field of global development governance by analyzing an academic deliberative space organized in parallel to the Fourth United Nations Conference on Financing for Development (FfD4). Drawing on critical development theory and critical perspectives on human rights, the study explores how expert debates respond to and reinterpret dominant framings of development financing as a neutral and technical set of instruments oriented toward efficient resource mobilization. Methodologically, the research combines qualitative discourse analysis with semantic network analysis, based on a systematically constructed corpus of recorded expert interventions and moderated debates produced during the academic event. The corpus includes contributions from diverse institutional actors participating in structured thematic panels. Using software-assisted coding (ATLAS.ti) and network analysis (UCINET), the study applies centrality measures (degree, closeness, and betweenness) to operationalize discourse as a relational structure. Analytical rigor was strengthened through iterative coding refinement, team-based review of categories, and structural robustness checks of the generated semantic networks. The findings reveal a hierarchically organized network in which concepts associated with cooperation, governance, sustainability, and innovation occupy structurally central positions, while narratives linked to social justice, community processes, and the expansion of rights remain comparatively peripheral. The findings suggest that development financing is predominantly framed through technocratic and procedural rationalities, while critical ethical perspectives circulate with more limited structural influence. By integrating discourse theory and social network analysis, the article contributes to sociological debates on global governance, power, and the organization of normative hierarchies in contemporary development.

## 1 Introduction

Development financing currently occupies a central position within the architecture of global governance. In a context marked by climate crisis, persistent inequalities, and geopolitical tensions, the key question is no longer only how much is financed, but how priorities are defined, which rationalities are considered legitimate, and which models of the future are enabled or excluded. Rather than operating as a purely technical and neutral mechanism, development financing can be understood as a political and institutional dispositif that structures power relations: it establishes criteria of eligibility, produces hierarchies of knowledge, and delineates the boundaries of what is considered “possible” in terms of public policy and territorial transformation. This perspective aligns with a growing body of research that has examined how development processes are increasingly shaped by financialised rationalities and technocratic governance frameworks. Existing studies have shown both how development is actively rendered investible through technical and financialised discourses, and how audit-based regimes of expertise contribute to framing development financing as a depoliticised domain of technical management (Taggart and Power, 2024; Tan, 2022).

Alongside the gradual displacement of public aid toward mechanisms such as private capital mobilization and blended finance, an expert language has been consolidated that tends to translate complex social problems into manageable categories (impact, efficiency, innovation, sustainability, accountability). This discursive shift is particularly significant because it not only accompanies instrumental changes but also reconfigures the normative framework through which development is interpreted. In this sense, analyzing development financing requires attention not only to instruments and financial flows, but also to the discourses through which they are legitimized, consensus is stabilized, and alternatives are rendered marginal or inaudible. This dynamic is closely linked to the rise of audit-based governance frameworks that frame development financing as a matter of technical expertise and accountability rather than political contestation (Tan, 2022).

This article is situated within critical development approaches and critical discourse theory in order to examine how expert discourse on development financing is structured within a specific deliberative space: the International University Conference on Financing for Development held at the Universidad Pablo de Olavide (Seville, Spain), organized in parallel to the United Nations International Conference on Financing for Development (July 2025). Drawing on the assumption that discourse shapes social reality, the study analyzes how certain notions acquire centrality and function as semantic infrastructures of the field, while others remain peripheral or depend on processes of translation in order to circulate.

While this study does not directly analyze the formal arenas of global development governance, it examines a situated academic deliberative space as a micro-institutional setting in which broader discursive logics of development governance are reproduced, translated, and negotiated. The relevance of this case therefore lies not in its representativeness of official UN negotiations, but in its capacity to reveal how dominant technocratic narratives permeate expert-academic discourse beyond formal institutional settings.

Methodologically, the study adopts a qualitative mixed-methods design combining (i) critical discourse analysis, (ii) semantic coding of the corpus in ATLAS.ti, and (iii) structural network analysis of conceptual co-occurrences through centrality measures (degree, closeness, and betweenness). This approach allows discourse to be operationalized as a relational structure—not as a collection of isolated opinions, but as a network in which concepts are connected, compete for centrality, and mediate tensions between instrumental rationalities and ethical-political concerns.

The article pursues a double objective. First, it seeks to identify which concepts occupy central positions and which semantic configurations organize expert discourse on development financing. Second, it critically interprets these regularities in light of a critical development framework and, in a complementary manner, through reference to Joaquín Herrera Flores’ Ethical Diamond as an interpretative lens for problematizing the relationships between development, power, and dignity. In doing so, the article contributes to a more nuanced understanding of development financing as a field of discursive contestation, showing how the semantic structure of discourse may reinforce dominant technocratic frameworks while simultaneously opening space for critical narratives oriented toward social justice and ecosocial transformation.

### 1.1 Critical development and the politics of development financing

The concept of development has historically been presented as a linear process of economic and social progress, associated with GDP growth and integration into global markets—what Wolfgang Sachs and Gilbert Rist conceptualize as hegemonic development, understood not merely as a set of policies but as a global ideology that naturalizes a particular worldview (Sachs, 1992; Rist, 2002). Since the mid-twentieth century, various critical traditions have challenged this conception, arguing that development does not constitute a neutral or universal horizon but rather a historical and discursive construction produced and reproduced through power relations, structural inequalities, and geopolitical hierarchies (Peet, 2002; Gautam, 2024).

From this perspective, development can be understood less as an objective response to poverty or global inequality than as a normative and performative language through which what is desirable, backward, and possible is defined, while specific forms of intervention, subordination, and dependency within the international system are legitimized (Ashrafuzzaman and Rahman, 2022). Critical development theories have highlighted how dominant models have frequently operated as mechanisms for reproducing dependency and inequality between the Global North and the Global South (Escobar, 1998). Rather than reducing structural gaps, development strategies based on economic liberalization, the attraction of foreign investment, and the commodification of commons have often reinforced historical asymmetries, eroding states’ economic sovereignty and weakening the social and community systems that sustain life in territories (Delgado-Baena, 2016). In this sense, development has often functioned less as a promise of emancipation than as a normative framework that defines legitimate priorities, orients public action, and justifies external interventions that reorganize local economies and subordinate the social sphere to objectives formulated from transnational arenas, thereby constraining situated agency and knowledge (Gumede, 2021; Eyben, 2012).

Within this context, development financing occupies a central position. Far from being merely a technical instrument for resource mobilization, development financing constitutes a structural mechanism of global governance. Through it, conditions, priorities, and eligibility criteria are established that delimit the margins of action available to states, social organizations, and territories (Bloom, 2024; Hilbrandt, 2025). The way development is financed—who provides resources, under what conditions, and through which control mechanisms—configures power relations that transcend the economic sphere and directly affect social organization, collective decision-making, and territorial autonomy (Bloom, 2024; Eyben, 2012).

From a critical perspective, development financing can be analyzed as a field in which diverse actors (multilateral financial institutions, states, cooperation agencies, the private sector, and civil society organizations) interact with unequal capacities to define agendas and normative frameworks (Ayodele, 2025; Bulfone et al., 2025). Within this field, financial capital, technical capital, and symbolic capital operate as strategic resources that enable certain actors to impose legitimate definitions of what counts as “effective,” “sustainable,” or “innovative” development. This dynamic tends to privilege standardized, measurable, and results-oriented approaches, often at the expense of complex, situated, and long-term social processes.

The growing centrality of instruments such as private resource mobilization, public–private partnerships, and so-called “blended finance” reinforces this logic. While these mechanisms are presented as pragmatic responses to the insufficiency of official development assistance, their expansion introduces criteria of profitability and risk management that reconfigure development priorities (Brown et al., 2023; Browne, 2022). In many contexts, this implies subordinating territories’ social and ecological needs to projects’ financial viability, displacing local communities from decision-making arenas and transforming social organizations into technical implementers of externally defined agendas (Bloom, 2024; Eyben, 2012).

In contrast to this dominant paradigm, various critical and ecosocial approaches have advocated alternative frameworks grounded in solidarity economies, social innovation, and community-based sustainability practices. These perspectives conceive development not as accumulation but as the expansion of collective capabilities, the reproduction of life, and the strengthening of social and territorial bonds (Delgado-Baena and Vela-Jiménez, 2022). From this standpoint, development financing should be oriented toward sustaining endogenous processes, respecting local economic systems, and recognizing the plurality of rationalities that coexist within territories, rather than imposing homogeneous models of growth and efficiency (Alarcón, 2020; Gumede, 2021).

Such alternatives tend to occupy peripheral positions within institutional frameworks of international financing, indicating that debates on development and its financing are not merely technical but profoundly political. Development financing thus emerges as a space of negotiation and contestation in which meanings, legitimacies, and models of the future are disputed (Nosrati et al., 2023). Understanding these dynamics therefore requires analyzing not only financial flows and instruments, but also the discourses, narratives, and power structures that sustain and operationalize them within global governance.

In this context, it becomes crucial to attend to the spaces from which alternative responses to these dynamics are produced, particularly when such responses emerge from territories and their social actors. From a critical perspective, territory cannot be understood merely as the site where development policies are implemented or financial flows are territorialized, but as a living configuration of social, institutional, and cultural relations that generates knowledge, meaning, and collective capacity for action (Torre, 2025; García-Madurga et al., 2020; Kato et al., 2022). Contemporary approaches emphasize that development processes acquire greater transformative density when territorial actors participate not only as beneficiaries but as subjects who interpret, resignify, and contest dominant intervention frameworks (Bastiaensen et al., 2021).

In this sense, territory becomes a political and epistemic space for the production of situated knowledge, care practices, and alternative forms of governance that critically challenge the vertical logics of traditional international cooperation. Recognizing this dimension entails shifting the analytical focus from the instrumental efficiency of resources toward the construction of collective capabilities, relational autonomy, and the formation of multi-actor alliances anchored in specific contexts. This shift is particularly relevant for social work and contemporary development approaches, insofar as it enables change to be understood not as an externally induced process, but as a co-constructed dynamic emerging from territories and observable in concrete practices of multi-actor articulation.

Despite these contributions, existing research has primarily examined development financing either as a structural mechanism of global governance or as a discursive field of power, but has paid more limited attention to how these dimensions are articulated within concrete expert deliberative spaces. In particular, there is a lack of empirical analyses that capture how meanings are relationally structured, stabilized, and contested through discourse in specific institutional contexts. This article addresses this gap by combining critical development perspectives with a relational analysis of discourse, focusing on how semantic structures organize and hierarchize expert understandings of development financing.

### 1.2 International conferences on financing for development and their critical-ecosocial implications

The cycle of international conferences on Financing for Development (United Nations, 2002, 2009, 2015, 2025) makes it possible to trace the evolution of global consensus regarding the financial means of development, as well as the persistent tensions between international cooperation, state sovereignty, and economic power (United Nations, 2002, 2009, 2015, 2025).

The United Nations (2002) resulted in the so-called Monterrey Consensus, which for the first time articulated an integrated vision of development financing based on shared responsibility between developed and developing countries. Within this framework, macroeconomic stability, private investment, and international trade were consolidated as central drivers of progress, progressively shifting emphasis from public aid toward the mobilization of domestic and market-based resources (United Nations, 2002).

The United Nations (2009), held in the context of the global financial crisis, reaffirmed the commitments established in Monterrey and incorporated demands for a more equitable international economic governance system, greater fiscal transparency, and sovereign debt relief mechanisms. However, its outcomes were constrained by the continuity of the dominant neoliberal paradigm and by the persistence of deep financial asymmetries among countries (United Nations, 2009).

In United Nations (2015), the Action Agenda expanded the discursive framework toward sustainable financing, explicitly linking the Sustainable Development Goals to South–South cooperation and to a greater role for public–private mechanisms. Despite this discursive shift, critical analyses have argued that the underlying structure of financial dependency remained largely unchanged (United Nations, 2015).

More recently, the United Nations (2025), concluded with the Seville Commitment to Action, introduced renewed language centered on global tax justice, financial resilience, and sustainability, with explicit emphasis on reforming the international economic architecture. Nevertheless, from a critical perspective, it has been noted that despite updated instruments and narratives, structural power logics continue to shape financial flows in ways that constrain the economic sovereignty of the Global South (United Nations, 2025).

Taken together, these international conferences on Financing for Development can be characterized by the progressive incorporation of discourses emphasizing global tax justice, sustainability, and financial resilience. However, from a critical-ecosocial paradigm, it remains necessary to interrogate the extent to which these proposals represent substantive transformations of the structural power relations that have historically shaped international cooperation. The literature suggests that development financing continues to operate as a mechanism of global governance that conditions the economic and political sovereignty of territories in the Global South (CEPAL, 2025; OECD et al., 2024).

From this perspective, emphasizing the relative autonomy of global regulatory organizations risks reifying the “global level” itself. As Scholte (2020) argues, “the global” can become constructed as an ontologically separate sphere of governance. Within such a framework, economic determinism narrows the concept of development as it relates to community-based and social organizations, whose own economic systems—often sustainable and territorially embedded—tend to be overlooked. The increasing orientation toward private capital mobilization, through instruments such as blended finance, tends to subordinate territorial strategies to logics of financial profitability, thereby reproducing historical inequalities between North and South (OECD, 2025).

In numerous communities across the Southern hemisphere, community-based intervention and development cooperation have historically centered on grassroots social organizations and collective decision-making systems, such as assemblies, communal councils, or long-standing cooperative practices such as the Andean minka. These organizational forms—grounded in mutual aid, reciprocity, collective labor, and solidaristic redistribution—constitute fundamental dispositifs of social and ecological sustainability (Gudynas, 2015).

One of the most visible effects of these dynamics is the financialization of nature, through which ecosystems and commons are integrated into global investment circuits, generating significant tensions in terms of climate and territorial justice (Dafermos, 2025). In community contexts, the channeling of resources through highly conditioned projects can weaken collective decision-making systems and reconfigure social organizations as technical implementers of externally defined agendas (Hasselskog, 2015).

For these reasons, development financing cannot be analyzed independently of the social organizational systems that sustain collective life in territories of the Global South. The recent United Nations (2025), particularly the proposals regarding reform of the international financial architecture, opens a debate that requires critical engagement. As recent institutional reports and analyses indicate, financial flows continue to reflect structural logics that condition territorial autonomy and reproduce persistent asymmetries between North and South (CEPAL, 2025; OECD et al., 2024).

### 1.3 Discourse, power, and global governance

Discourse analysis constitutes a central tool for understanding power dynamics in contemporary global governance, particularly in domains such as development financing, where narratives, legitimacies, and institutional frameworks shape public action. From a Foucauldian perspective, discourse is not merely a vehicle of information but a producer of social reality, insofar as it establishes regimes of truth that determine which problems are recognized, how they are interpreted, and which solutions are considered legitimate (Foucault, 1970, 1972). In this sense, discourse delineates the boundaries of what can be said, institutes hierarchies among actors and forms of knowledge, and generates structured silences that exclude alternative ways of knowing and inhabiting the world.

Critical Discourse Analysis (CDA), particularly in the formulations of Fairclough, N. and van Dijk, T., further deepens this understanding of language as a social practice constitutive of structures of domination. Fairclough (1992, 2013) emphasizes that institutional discourses produce and reproduce hegemonic relations that naturalize technocratic models of governance, especially within the field of global development. In parallel, van Dijk (1993, 1998) highlights how discourses shape collective mental models through which political and economic actors interpret reality, contributing to processes of ideological reproduction that may reinforce dominant elite interests while delegitimizing subaltern perspectives. This conception is particularly relevant for analyzing how multilateral financial institutions configure criteria, languages, and evaluative frameworks in development financing.

The cognitive dimension of discourse has been further expanded by recent studies examining how dominant narratives establish shared interpretative frameworks. Authors such as Kress (2009) and Reisigl (2017) argue that institutional discourses influence how social actors understand what is viable, desirable, or possible in the realm of development policy. More recent contributions in Critical Discourse Studies, particularly corpus-assisted approaches, have further demonstrated how discursive strategies and linguistic patterns contribute to the construction and transformation of political narratives across contexts (Lu and Yu, 2024; Lu, 2026). In the field of international financing, such frameworks tend to privilege standardized and replicable solutions, often limiting attention to local contexts and situated knowledge.

Critical scholarship on global governance has also demonstrated how discourses of sustainable development and climate financing may reproduce extractivist and anthropocentric logics, even when adopting apparently inclusive or environmentally responsible language (Brand, 2021). From an ecosocial perspective, these approaches are problematic insofar as they tend to prioritize global economic imperatives over social and ecological well-being. In this way, discourses on development financing do not merely reflect economic priorities but actively contribute to consolidating governance models centered on efficiency, impact measurement, and accountability, frequently displacing participatory and community-oriented approaches.

Building on these theoretical foundations, recent research has shown how dominant narratives in global governance reproduce specific configurations of power that privilege economistic, metric-driven, and depoliticized approaches to sustainable development. These dynamics are particularly visible in the field of development financing, where multilateral organizations shape narratives that orient investment priorities and condition the public action of states in the Global South. Taken together, the integration of the contributions of Foucault, Fairclough, and van Dijk with contemporary scholarship allows us to understand that discourses of global governance do not merely represent the reality of development; they actively participate in producing it, generating hierarchies among actors and defining silences that shape the contours of development financing.

Within this framework, discourse analysis becomes a fundamental tool for critical social sciences, enabling the identification of how hierarchies of actors are constructed, how particular rationalities are legitimized, and how structured silences are produced. The integration of contemporary CDA approaches with critical development perspectives makes it possible not only to understand the discursive mechanisms of global governance, but also to critically interrogate the normative horizons that orient models of development financing and their implications for democratic, situated, and ecosocially responsible forms of governance.

Taken together, these perspectives inform the analytical strategy of this study. Rather than adopting a single strand of Critical Discourse Analysis, the article draws on a broader Critical Discourse Studies (CDS) approach (Wodak and Meyer, 2016; Flowerdew and Richardson, 2018), integrating insights from Foucauldian discourse theory, Fairclough’s understanding of discourse as social practice, and van Dijk’s focus on cognitive and ideological structures. This combined perspective allows us to analyze development financing discourse simultaneously as a regime of truth, a field of power-laden social practices, and a structured semantic network. In doing so, the study connects critical development theory with discourse analysis and network-based approaches, making explicit how these frameworks are articulated within a unified analytical strategy.

### 1.4 Discursive and semantic networks as analytical frameworks

The analysis of discursive and semantic networks has become an important analytical approach for understanding how meanings are produced, circulated, and contested within the fields of development and global governance. From a Foucauldian perspective, networks of statements can be understood as discursive formations that establish regimes of truth and delimit the frameworks within which certain forms of knowledge become legitimate while others remain marginalized or rendered invisible (Foucault, 1972). These networks do not develop linearly; rather, they are organized through constellations of signifiers, concepts, and metaphors that form relatively stable structures of meaning.

Within such formations, particular nodes may acquire central positions that stabilize dominant interpretations of concepts such as sustainability, governance, or financing. In this regard, van Dijk (1993, 1998) argues that discursive centrality often corresponds to the capacity of dominant institutional actors to stabilize meanings through strategies of repetition, institutionalization, and exclusion. Similarly, Fairclough (1992, 2013) maintains that discursive networks reproduce unequal social relations insofar as dominant nodes naturalize technocratic approaches and reduce socioterritorial complexity to standardized frameworks.

Recent research further confirms the political dimension of discursive networks, showing how international alliances are structured around narrative networks that consolidate hierarchies between global actors and peripheral territories, as Bexell and Jönsson (2024) argue. The peripheries of such networks may contain alternative or critical signifiers that, although less institutionalized, constitute sites of discursive contestation and potential reconfiguration. At the macro level, Scholte (2020) suggests that mapping these networks allows for the identification of how global institutions exercise multidimensional power through linguistic configurations that structure decision-making processes.

Within this framework, the notion of centrality becomes crucial for understanding how discourses are structured and hierarchized in global governance. Drawing on classical contributions to social network analysis, Freeman (1978) and Freeman (1977) demonstrated that the position occupied by nodes within a network is not neutral but reflects different degrees of visibility, influence, and articulatory capacity. Transposed to discourse analysis, this insight implies that concepts, actors, or narratives occupying central positions possess greater capacity to define legitimate interpretative frameworks, whereas those located in peripheral positions tend to be subordinated, marginalized, or rendered less visible. Centrality, therefore, does not refer merely to frequency or quantitative presence, but to a form of relational power emerging from the structure of the network itself.

From a critical perspective, this approach is particularly pertinent for analyzing development financing as a discursive field. It enables the identification of how certain signifiers (such as efficiency, sustainability, impact, or innovation) consolidate as hegemonic cores of discourse, articulating consensus and delimiting the boundaries of what can be legitimately expressed. At the same time, examining network peripheries makes it possible to render visible alternative narratives, situated knowledges, and ecosocial perspectives that, while present, lack the structural capacity to reconfigure the dominant frame. In this way, the incorporation of network analysis and centrality as analytical categories offers a conceptual bridge between critical discourse theory and empirical investigation, operationalizing the relationships among discourse, power, and global governance.

Taken together, this framework allows for the mapping of cores of meaning, zones of contestation, and critical peripheries, providing a deeper understanding of how discourses are structured, legitimized, and transformed in global contexts.

### 1.5 Ethical dimensions of development financing

From critical theoretical perspectives, development financing cannot be understood solely through instruments or measurable outcomes, but must be examined as an ethically and politically contested field. The Ethical Diamond proposed by Herrera Flores functions here as a normative interpretative framework rather than a methodological tool (Herrera Flores, 2008). Rooted in a conception of human rights as historical processes of struggle, the model highlights the relational tensions between symbolic, material, and institutional dimensions of social life.

Unlike descriptive cultural models such as Griswold’s cultural diamond (GRISWOLD, 2005), the Ethical Diamond has an explicitly critical orientation, aimed at revealing how power relations shape which discourses become visible and legitimate. In the field of development financing, decisions often framed as technical are embedded in political rationalities and institutional hierarchies that delimit participation and define priorities (Khosla et al., 2025; Genovese and Hermida-Rivera, 2022).

Previous research has applied this framework to public policy and territorial development processes (Baena and Abellán, 2016; Delgado-Baena et al., 2024; Bellerín, 2017). Consistent with this tradition, the present study employs the Ethical Diamond as a critical lens to interpret discursive configurations and power asymmetries shaping development governance (Delgado-Baena, 2016; Kentikelenis et al., 2023; Hickel et al., 2022; Bigger and Webber, 2023).

## 2 Materials and methods

### 2.1 Research design

This study adopts a qualitative mixed-methods research design aimed at analyzing how expert discourse on development financing is structured, circulates, and is reproduced. The approach integrates three complementary analytical layers: critical discourse analysis, inductive–deductive semantic coding, and co-occurrence network analysis. Rather than being conceived as independent techniques, these layers form a unified analytical strategy designed to capture both the semantic content of discourse and its relational organization, as well as its ethical and political implications.

First, critical discourse analysis is employed to examine the semantic, normative, and political dimensions that permeate debates on development financing, paying attention to the assumptions, hierarchies, and rationalities structuring expert interventions (Frawley, 1993). Second, the corpus undergoes a systematic semantic coding process combining an inductive logic—aimed at identifying emergent categories—with a deductive component informed in part by the Ethical Diamond framework (Herrera Flores, 2008). Finally, a network analysis of conceptual co-occurrences is conducted in order to operationalize discourse as a relational structure, using the classical centrality measures of degree, closeness, and betweenness proposed by Freeman (1978).

The objective of this methodological strategy is to identify how meanings circulate within expert communities, which concepts acquire positions of discursive centrality, and how ethical and political tensions are articulated within deliberative spaces linked to development financing. Conceiving discourse as a relational configuration—rather than as an aggregate of isolated opinions—makes it possible to render visible both dominant narratives and marginal or emerging perspectives that contest hegemonic understandings of development.

This research design draws on structural sociology (Freeman, 1978), critical discourse theory (Frawley, 1993), and critical development and human rights studies (Herrera Flores, 2008), assuming that development financing does not constitute a merely technical domain, but a field traversed by power relations, institutional arrangements, and ethical disputes. From this perspective, the integrative strategy adopted here enables a deeper understanding of how expert actors produce, negotiate, and legitimize meanings within multilateral agendas and contemporary processes of territorial governance.

### 2.2 Data collection

Data collection was conducted during the International University Conference on Financing for Development held at the Universidad Pablo de Olavide (Seville, Spain). The event was organized within the framework of an international development cooperation program involving public institutions and academic actors and took place in parallel to the United Nations International Conference on Financing for Development held in the same city in July 2025.

The conference brought together experts from governmental agencies, multilateral organizations, local authorities, and civil society organizations. The event was structured around four thematic panels, each dedicated to a key dimension of development financing, thereby enabling the capture of diverse institutional positions, scales of intervention, and interpretative frameworks.

The composition of the thematic panels and the distribution of coded interventions are summarized in Table 1.

**Table 1:** Description of thematic panels and number of coded interventions.

Panels	Main theme	No. of panelists	No. of moderators	Types of actors represented
Panel 0	Opening narrative on financing	2	1	Journalist, international expert
Panel 1	Opening keynote: “Territory, development and solidarity”	1	1	Social cooperatives, international expert
Panel 2	Cooperation, public policies and new governance	4	1	Academia, public administration
Panel 3	Cooperation and territory	4	1	Academia, cooperatives, public administration
Panel 4	Civil society	4	1	Civil society organizations, NGOs

As shown in Table 1, the panels reflect institutional diversity across governance levels and organizational types, providing a heterogeneous empirical foundation for the subsequent semantic and relational analyses.

The analytical corpus consists of three types of materials: (a) audio-recorded oral interventions by panelists and moderators; (b) full transcripts of these interventions, complemented by field notes produced during the event; and (c) supplementary documentation, including official programs, institutional presentations, and other publicly available materials. All audio material was transcribed verbatim and subsequently refined to remove repetitions, hesitations, and non-substantive expressions, while preserving the semantic and argumentative content of the discourse. The resulting corpus constitutes a coherent and sufficiently robust empirical basis for systematic semantic coding and network analysis.

The analysis was conducted in accordance with the ethical principles of the participating institutions. As all interventions took place in a public academic setting and participants acted in their professional capacities, the study relied exclusively on publicly accessible material and focused on aggregated discursive patterns rather than individual-level data. No personal or sensitive information was collected, processed, or analyzed.

### 2.3 Coding procedures

The complete corpus of the International Conference was analyzed through a coding process in ATLAS.ti 25 conceived as an interpretative analytical process aimed at capturing the semantic emergence of expert discourse. The deductive dimension did not operate at the coding stage but rather informed the subsequent theoretical interpretation of relational discourse structures, drawing on critical frameworks on power, governance, and human rights.

The coding process was guided by a semantic-discursive analytical framework, in which codes were defined as meaningful units capturing recurrent concepts, problem framings, and interpretative categories mobilized by participants in relation to development financing. Rather than coding merely descriptive topics, the analysis focused on how key notions (e.g., governance, sustainability, cooperation, inequality) were articulated, associated, and endowed with meaning within expert discourse. In this sense, coding was oriented toward identifying both thematic content and underlying discursive rationalities.

The inductive component was operationalized through open coding of the corpus, allowing categories to emerge from the data based on semantic recurrence and interpretative relevance. These initial codes were progressively refined through axial coding, grouping them into broader conceptual clusters. The deductive dimension did not involve the imposition of predefined categories at the coding stage, but rather informed the subsequent interpretation of the resulting semantic structures, particularly in relation to questions of power, governance, and normative tensions.

Coding was primarily conducted by the first author, with periodic review and discussion of emerging categories within the research team to ensure interpretative consistency. In line with Critical Discourse Studies epistemology, reliability was not approached through statistical inter-coder agreement, but through iterative refinement, intra-coder consistency checks, and team-based interpretative validation, emphasizing coherence, transparency, and theoretical grounding of the analytical process.

The theoretical interpretation of the identified discursive patterns was supported by Joaquín Herrera Flores’ Ethical Diamond, not as a coding scheme or classificatory scaffold, but as a normative interpretative lens. From this perspective, the semantic and relational regularities of the discourse were examined in relation to broader dimensions linked to experiences, rationalities, practices, and institutions, as well as to tensions between semantic and material axes of development. This approach enabled an ethically informed contextualization of emerging discursive structures without imposing external analytical categories during the coding process.

Building on this analytical framework, the coding process identified unanticipated concepts, narrative tensions, and emergent semantic patterns directly from participants’ interventions. Through successive rounds of revision and axial coding, these codes were refined into a consolidated analytical system comprising 121 codes, subsequently grouped into thematic clusters associated with notions such as development, governance, cooperation, conflict, inequality, and institutional critique. This procedure preserved the expressive richness of the discourse and captured its semantic complexity without superimposing predefined analytical categories onto the corpus.

To ensure accessibility and transparency of the original Spanish coding scheme, a bilingual codebook (Spanish–English) of the 121 analytical categories is provided in Supplementary file.

Prior to relational analysis, the coding system underwent a process of terminological refinement and standardization, involving the elimination of redundancies and the merging of overlapping codes. An intra-coder reliability check was conducted through partial recoding of the corpus after a temporal interval and systematic comparison of discrepancies in order to ensure consistency in the application of the analytical framework (Miles et al., 2014). The result was a robust and internally coherent coding system suitable for supporting conceptual co-occurrence analysis and subsequent relational interpretation.

Based on this refined structure, co-occurrence matrices were generated using the ATLAS.ti co-occurrence explorer. These matrices, exported in .csv format, enabled the identification of systematic conceptual co-presence within the same textual segments and constituted the empirical basis for the network analysis developed in the following sections.

In a complementary manner, the final codes were placed in dialogue with the normative framework of critical development as an interpretative reference. This articulation was not employed as a classificatory structure or as a mode of findings presentation, but as a transversal ethical-critical orientation informing the relational reading of the discursive network and the identification of emergent ethical and political tensions.

### 2.4 Network construction and analysis

#### 2.4.1 Generation of co-occurrence matrices

The relational analysis of discourse was based on the construction of a co-occurrence matrix generated in ATLAS.ti 25. This matrix records the frequency with which two codes are jointly activated within the same textual segment of the corpus, treating such simultaneity as an indicator of conceptual proximity or semantic resonance between analytical categories. The resulting structure takes the form of a square n × n matrix, in which rows and columns represent the final set of codes and each cell contains the absolute frequency of co-occurrence between the corresponding pair of codes.

In order to focus the analysis on structurally meaningful relationships, a reduced subset of the most intense co-occurrences was selected (top 25 weighted code pairs). This threshold was established through an exploratory analytical criterion aimed at identifying conceptually dense relational linkages while avoiding excessive dispersion within the semantic network. By concentrating on the strongest connections, the analysis emphasizes the core relational architecture of expert discourse rather than peripheral or weakly connected associations.

#### 2.4.2 Construction of the weighted network

Based on the co-occurrence matrix, a semantic network was constructed in which codes function as nodes and co-occurrences as weighted edges proportional to their observed frequencies. In order to enhance analytical readability and reduce noise associated with marginal relationships, low-intensity ties were filtered out, retaining only those edges whose weights were situated above the 75th percentile of the frequency distribution. This thresholding procedure allowed the analysis to focus on structurally significant conceptual associations.

The resulting network was visualized using force-directed layout algorithms and subjected to descriptive network analysis. The metrics computed included degree centrality, weighted degree, and modularity. These measures enabled the identification of structurally central nodes, codes performing bridging or intermediary functions, and the emergence of thematic subcommunities within the network. The detected structural patterns were subsequently interpreted in light of the study’s analytical framework.

The final network consisted of 121 nodes and 104 weighted edges after applying the 75th percentile threshold. The application of this filtering procedure allowed the retention of the most structurally significant relationships while reducing noise and improving the interpretability of the network structure. Community detection was conducted using modularity-based clustering procedures implemented in UCINET 6, following standard partitioning approaches for weighted networks, resulting in the identification of distinct thematic groupings within the network.

Although modularity-based clustering was applied, the study does not rely on the modularity score (Q) as a primary validation criterion. Instead, in line with the mixed-methods design, cluster interpretation is grounded in the convergence between structural patterns and qualitative discourse analysis, prioritizing interpretative coherence over purely metric validation.

#### 2.4.3 Structural network analysis (UCINET)

The refined co-occurrence matrix was analyzed using UCINET 6, applying the classical centrality measures proposed by Freeman (Freeman, 1978). This approach has been widely employed in relational and discourse-oriented research (De las Heras et al., 2021) and enabled a systematic examination of the structural configuration of the semantic network, as well as the relative position occupied by each concept within it.

Three complementary centrality indicators were computed. Degree centrality was used to assess the discursive prominence of each concept, measured by the number of direct ties it maintains with other nodes in the network. Closeness centrality was calculated to identify structurally strategic concepts located at shorter average path distances from all other nodes, thereby indicating their relative accessibility within the overall network configuration. Unlike degree centrality, which captures local visibility, closeness reflects the structural capacity of a concept to access and connect multiple thematic areas across the network.

Betweenness centrality was estimated to detect nodes that function as bridges between otherwise weakly connected or distinct semantic clusters. This measure identifies concepts that occupy intermediary structural positions and that facilitate the circulation of meanings across different discursive domains. Rather than reflecting mere frequency or thematic prominence, betweenness centrality captures the extent to which a node mediates relational pathways within the network.

Together, these measures provide a structural basis for examining discursive hierarchies, conceptual dependencies, and patterns of semantic articulation, which are analyzed in detail in the Results and findings section.

#### 2.4.4 Semantic interpretative layer

In order to avoid a purely metric reading of the network structure, the structural analysis was complemented by a detailed qualitative examination of the quotations associated with codes occupying central positions within the network. This phase enabled the contextualization of centrality indicators through close attention to the concrete discursive content, focusing on how concepts were mobilized, articulated, and endowed with meaning by participants.

Through this integrative procedure, analytically relevant semantic clusters—such as development and change, cooperation, governance, and ethical tensions—were identified. These clusters were not treated as predefined categories but emerged from the interaction between structural metrics and qualitative interpretation. The integration of structural network analysis and qualitative reading enabled a critically informed interpretation of expert discourse, oriented toward understanding not only which concepts occupy central positions, but also the rationalities underpinning their articulation within the discursive field.

The overall analytical process of the study is summarized in Table 2.

**Table 2:** Phases of the research design and analytical procedure.

Phase	Analytical stage	Main procedures
Phase 1	Research design and data collection	Qualitative mixed-methods design Selection of the empirical field Recruitment of expert participants Recording and transcription of sessions Construction of the analytical corpus
Phase 2	Corpus processing (ATLAS.ti)	Development of a coding framework Open and axial coding Identification of emerging categories Refinement and validation of codes Analytical organization of categories
Phase 3	Preparation for relational analysis	Generation of co-occurrence matrices Extraction of code relationships Grouping of analytical categories Preparation of weighted matrices
Phase 4	Network analysis and interpretation	Construction of semantic networks Structural analysis (centrality, clustering) Identification of key nodes Qualitative interpretation Integration of findings

#### 2.4.5 Analytical validation and methodological rigor

To ensure analytical robustness and credibility, we implemented a multi-level validation strategy consistent with qualitative research standards regarding significance, sufficiency, and transparency (Lincoln and Guba, 1985; Stenius et al., 2017; Strauss and Corbin, 1998).

#### 2.4.6 Corpus significance and contextualization

The empirical corpus derives from academic workshops designed and organized by the research team as part of an Education for Development project at the University Pablo de Olavide. Rather than an opportunistic event, these workshops formed part of the research design itself and functioned as a structured deliberative space linked to the Fourth International Conference on Financing for Development held in Seville.

As the deliberative space was designed by the research team, it is also important to acknowledge that this design may have influenced the discursive configurations observed. In line with Critical Discourse Studies, this co-production of discourse is understood as part of the analytical context rather than an external bias to be eliminated.

Participants were selected based on institutional relevance and territorial diversity. Invitees included individuals directly involved in the UN FfD process or with recognized academic and professional expertise in development financing. The workshops brought together actors from Latin America, Europe, and Asia, representing academia, public administration, and civil society (see Table 1). The aim was not to steer interventions but to facilitate structured deliberation among heterogeneous expert positions within the field of development governance.

This setting operated as a micro-institutionalized field of expert discourse at a specific political juncture, allowing findings to be situated within their socio-political conditions of production (Hammersley, 2018; Stenius et al., 2017).

#### 2.4.7 Iterative coding and internal coherence

Coding was conducted inductively in ATLAS.ti following an iterative refinement process. AI-assisted features were used only as exploratory support under a human-in-the-loop approach: all automated suggestions were manually reviewed, modified, or discarded. The final coding structure resulted exclusively from researcher-led interpretive decisions grounded in the corpus.

After initial open coding, categories were reorganized through successive rounds of refinement, merging redundancies and clarifying conceptual boundaries. A partial recoding phase was conducted after stabilization to assess intra-coder consistency (Miles and Huberman, 1994) Discrepancies were resolved through systematic comparison of coding criteria and conceptual clarification.

Double coding with statistical agreement coefficients was not implemented, in line with Critical Discourse Analysis epistemology, which conceives meaning as relational and situated. Reliability was therefore understood as interpretive stability and categorical coherence across the analytical process.

#### 2.4.8 Team-based interpretive validation

Emergent categories and patterns were periodically reviewed within the research team. These discussions involved close examination of textual segments, alternative readings, and explicit justification of coding decisions. This procedure functioned as investigator triangulation, enhancing credibility (Erlandson et al., 1993) and reducing individual interpretive bias.

#### 2.4.9 Theoretical saturation and analytical sufficiency

The coding system was considered stabilized when successive iterations yielded no new substantive categories, but only minor refinements. In later stages, additional segments reinforced existing categories rather than expanding the analytical framework. Recurring patterns across panels and actor profiles indicated theoretical saturation (Lincoln and Guba, 1985; Strauss and Corbin, 1998).

Sufficiency is understood here as conceptual sufficiency: the analysis reconstructed the semantic architecture of the deliberative space without significant structural alteration upon incorporation of additional data.

#### 2.4.10 Structural robustness of the network analysis

To avoid overinterpreting isolated associations, a minimum co-occurrence threshold (*f* ≥ 5) was applied when constructing the semantic network in UCINET. This threshold was defined after examining the frequency distribution of co-occurrences to balance relational density and structural clarity, ensuring that only recurrent patterns were retained.

Degree, closeness, and betweenness centrality (Freeman) were interpreted comparatively rather than in isolation. Structurally central nodes were qualitatively cross-checked against their supporting textual fragments, preventing purely formal readings of network outputs and ensuring alignment between structural prominence and discursive relevance.

#### 2.4.11 Analytical triangulation

Validation relied on the articulation of three complementary levels: Critical Discourse Analysis (interpretive), systematic coding (categorical), and relational modeling (structural). Findings were considered robust when convergence emerged between textual recurrence, categorical coherence, and structural centrality. This triangulation integrates qualitative interpretation with relational evidence within a coherent analytical framework.

In sum, these procedures ensure procedural transparency, internal coherence, and structural consistency in the study’s conclusions. Table 3 summarizes the analytical validation procedures implemented to strengthen methodological rigor and ensure the robustness of the findings.

**Table 3:** Analytical validation procedures and reliability strategies.

Validation dimension	Procedure implemented in this study	Analytical purpose
Corpus significance	Structured deliberative research design; selection of participants with prior involvement in the UN FfD process; territorial and institutional diversity	Ensure socio-institutional relevance and contextual grounding of discourse
Coding process	Inductive coding in ATLAS.ti; human-in-the-loop review of AI-assisted suggestions; iterative refinement of categories	Maintain interpretive control and semantic coherence
Intra-coder consistency	Partial recoding after stabilization phase; revision of category boundaries and decision rules	Strengthen internal reliability of the coding system
Team-based validation	Periodic analytical discussions; contrast of alternative interpretations; corpus-based justification of decisions	Reduce individual interpretive bias and enhance credibility
Theoretical saturation	Stabilization of categories; redundancy of patterns across panels and actor profiles	Ensure conceptual sufficiency of the dataset
Network robustness	Co-occurrence threshold (*f* ≥ 5) defined after frequency distribution examination; comparative interpretation of degree, closeness, and betweenness centrality	Prevent structural artefacts and overinterpretation of marginal ties
Analytical triangulation	Convergence between textual recurrence, categorical coherence, and structural centrality	Integrate qualitative interpretation with relational evidence

## 3 Results and findings

### 3.1 General structure of the discursive network

The co-occurrence analysis reveals a moderately decentralized semantic network characterized by the presence of a defined discursive core and several thematic poles connected with varying intensity. The overall degree centralization of the network (approximately 4.6%) indicates a relatively low concentration of structural prominence, suggesting that expert discourse on development financing is not organized around a single dominant concept but rather around multiple interconnected nodes.

The network visualization confirms this polycentric structure. A compact central area emerges, composed of highly interconnected concepts associated with cooperation, learning and collaboration, financing and governance, sustainability, and innovation. These nodes display dense relational ties and occupy structurally central positions within the network.

Surrounding this core, several less densely connected clusters can be identified. These include concepts related to social inequality, rights and equity, and critical reflection. Although these nodes remain connected to the central structure, their lower relational intensity and more peripheral positioning indicate a comparatively reduced structural prominence within the network.

Overall, the semantic network presents a structured yet distributed configuration, combining a dense relational nucleus with peripheral thematic areas linked through weaker but still meaningful connections.

**Figure 1:**
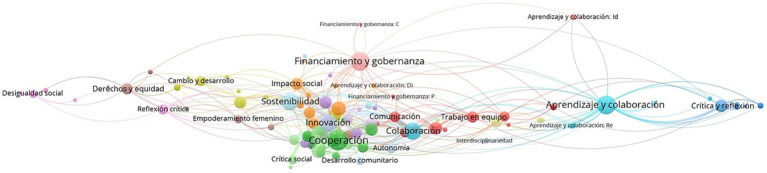
Semantic co-occurrence network of development financing discourse. Network visualization showing the structural configuration of the semantic co-occurrence analysis. Node size reflects degree centrality. Colors represent modular clusters identified through network clustering analysis. Edge thickness corresponds to the strength of co-occurrence between concepts. Node labels are presented in Spanish, as the empirical corpus consisted of interventions originally delivered in Spanish.

The graphical representation of the network is presented in Figure 1.

Node labels are presented in Spanish to preserve the original empirical coding. A full bilingual codebook (Spanish–English) is available in Supplementary file.

### 3.2 Degree centrality: structurally prominent concepts

The results of the degree centrality analysis reveal an uneven distribution of discursive prominence within the semantic network. The concept cooperation emerges as the most central node (47; normalized degree = 5.595), indicating that it maintains the highest number of direct connections with other concepts in the network.

At a second level of centrality, learning and collaboration (41; 4.881) and funding and governance (39; 4.643) occupy closely positioned values. The proximity of these scores suggests the presence of a relatively compact central configuration composed of interconnected procedural, relational, and governance-oriented concepts.

Other concepts such as innovation (34; 4.048), collaboration (33; 3.929), and sustainability (33; 3.929) also display relatively high degree values, indicating their integration within the main relational structure of the network.

In contrast, concepts associated with more explicitly normative dimensions show substantially lower degree centrality scores. Human rights (9; 1.071), social justice (12; 1.429), and social inequality (8; 0.952) occupy more peripheral structural positions in terms of direct connectivity. From a discourse-analytical perspective, this uneven distribution of centrality reflects the differential capacity of concepts to structure the field of meaning. The prominence of cooperation, governance, and innovation suggests the consolidation of a technocratic-discursive framework in which development financing is constructed as a matter of coordination and efficiency, while normative concepts such as human rights and social justice are positioned in ways that limit their capacity to shape dominant interpretations.

At the aggregate level, the network presents a low overall degree centralization (4.638%), indicating that discursive connectivity is distributed across multiple nodes rather than concentrated in a single dominant concept. Nevertheless, a limited number of nodes concentrate a comparatively higher share of direct ties, reinforcing the relative prominence of technocratic and operational signifiers within the overall structure.

Table 4 presents the five concepts with the highest degree centrality.

**Table 4:** Top five concepts by degree centrality.

Rank	Concept	Degree	Normalized degree
1	Cooperation	47	5.595
2	Learning and collaboration	41	4.881
3	Funding and governance	39	4.643
4	Innovation	34	4.048
5	Sustainability	33	3.929

**Figure 2:**
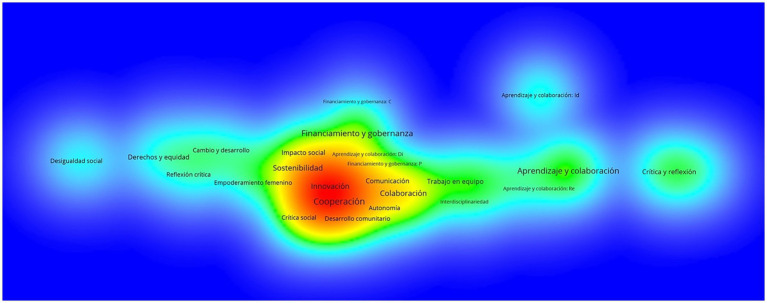
Heatmap representation of degree centrality values. Heatmap illustrating the relative intensity of degree centrality across key concepts in the semantic network. Darker tones indicate higher levels of structural prominence. Concept labels correspond to the original Spanish coding framework derived from the empirical corpus.

Figure 2 provides a heatmap representation of degree centrality values, visually illustrating the differential intensity of relational prominence across concepts in the network.

Node labels are presented in Spanish to preserve the original empirical coding. A full bilingual codebook (Spanish–English) is available in Supplementary file.

### 3.3 Closeness centrality: structurally strategic nodes

Closeness centrality values show a differentiated distribution across the semantic network, with normalized scores ranging from 36.04 to 20.24 (M = 27.69; SD = 2.82).

At the top of the distribution, funding and governance (36.036; farness = 333) occupies the highest closeness value, indicating that it is, on average, at shorter path distances from all other nodes in the network. This structural position suggests a high level of accessibility within the overall discursive configuration.

It is followed by learning and collaboration (34.582; farness = 347) and citizen participation (33.241; farness = 361), which also display relatively short average distances to the rest of the network. These concepts are therefore positioned in structurally advantageous locations that facilitate connections across multiple thematic areas.

Additional concepts with comparatively high closeness scores include research (33.058; farness = 363), sustainability (32.967; farness = 364), social impact (32.880), and innovation (32.520). Their proximity to other nodes indicates that they occupy positions enabling relatively efficient relational access within the network structure.

By contrast, concepts such as human rights (28.30), social justice (29.06), and social inequality (between 25.26 and 20.24) present lower closeness values and higher farness scores (up to 593), reflecting greater average structural distance from the rest of the network.

Table 5 presents the five concepts with the highest closeness centrality values.

**Table 5:** Top five concepts by closeness centrality.

Rank	Concept	Closeness	Farness
1	Funding and governance	36.036	333
2	Learning and collaboration	34.582	347
3	Citizen participation	33.241	361
4	Research	33.058	363
5	Sustainability	32.967	364

Overall, the closeness analysis indicates that a limited group of concepts occupies structurally strategic positions characterized by shorter relational distances, whereas other concepts are positioned at greater structural remove within the semantic network.

### 3.4 Betweenness centrality: bridging nodes and structural mediation

Betweenness centrality identifies concepts that play a structurally strategic role in network articulation, not because of their frequency or thematic prominence, but because of their capacity to connect otherwise differentiated semantic clusters. This measure highlights nodes through which a substantial proportion of shortest paths between concepts are routed, indicating their intermediary positioning within the discursive network.

The results show that learning and collaboration emerges as the primary bridging node in the network, with a non-normalized betweenness value of 1950.64 and a normalized value of 27.320. It is closely followed by funding and governance, with a betweenness of 1896.85 (normalized 26.567). These two nodes concentrate the bridging function in a pronounced manner, channeling a substantial share of geodesic paths connecting multiple thematic areas of the discourse.

Notably, several concepts display relevant betweenness values despite not occupying the most central positions in terms of degree. Rights and equity presents a betweenness value of 682.67 (normalized 9.561), followed by sustainability (480.77; normalized 5.733) and critical reflection (413.88; normalized 5.797). This pattern indicates that these concepts function as intermediary connectors between the core relational structure and more peripheral thematic areas, linking otherwise weakly connected parts of the network.

From a Critical Discourse Studies perspective, this intermediary position can be interpreted as a mechanism through which certain discursive elements mediate and translate heterogeneous meanings into governance-compatible frameworks. Concepts such as learning and collaboration operate not only as structural bridges but also as discursive devices that facilitate the circulation of narratives while potentially neutralizing more conflictive or redistributive interpretations.

Table 6 presents the five concepts with the highest betweenness centrality values.

**Table 6:** Top five concepts by betweenness centrality.

Rank	Concept	Betweenness	Normalized betweenness
1	Learning and collaboration	1,950.64	27.320
2	Funding and governance	1,896.85	26.567
3	Rights and equity	682.67	9.561
4	Sustainability	480.77	5.733
5	Critical Reflection	413.88	5.797

This pattern is further supported by the network betweenness centralization index, which reaches 26.16%, indicating a moderate-to-high concentration of mediation capacity in a limited number of nodes. This result suggests that the structural conditions for connectivity across the discursive network are not evenly distributed, but are influenced by the intermediary positioning of a small set of key concepts.

### 3.5 Semantic clusters and ethical reading of the discourse

The integration of structural network analysis with the qualitative examination of quotations associated with central codes made it possible to identify several relatively coherent semantic clusters organizing expert discourse on development financing around differentiated rationalities.

First, a dominant cluster emerges around cooperation, learning and collaboration, and related procedural categories. This semantic constellation displays high internal density and strong connectivity with other areas of the network, suggesting that it functions as a primary organizing framework of the discourse. The quotations associated with this cluster emphasize processes, methodologies, institutional coordination, and relational capacities, constructing a narrative in which cooperation is framed predominantly as a technical and operational practice rather than as a site of political contestation.

This framing can be understood as a discursive strategy that contributes to the depoliticization of development financing, as it foregrounds procedural and relational dimensions while backgrounding structural inequalities and power asymmetries. In this sense, cooperation is not only a central concept, but also a discursive construction that stabilizes a consensual and non-conflictive representation of development processes.

A second relevant cluster is articulated around financing, governance, and sustainability, exhibiting a more explicitly technical-normative profile. This group connects debates on financial architecture, policy instruments, and regulatory frameworks with references to sustainability goals and performance-oriented criteria. Structurally, this cluster operates as a stabilizing bridge between technical language and normative claims, contributing to the consolidation of an expert vocabulary that translates complex social and territorial challenges into manageable and governance-compatible categories.

Alongside these dominant nuclei, a less dense but analytically significant critical cluster can be identified, associated with notions such as rights and equity, social inequality, and critical reflection. Although this cluster occupies a more peripheral position within the network, its presence introduces explicit questioning of power asymmetries, redistributive effects of financing mechanisms, and the ethical limits of the prevailing development model.

From an ethical reading informed by critical development perspectives, the uneven articulation of these clusters reflects a structural tension between instrumental rationalities—centered on efficiency, coordination, and incremental innovation—and ethical-political rationalities focused on redistribution, recognition, and social justice (Table 7).

**Table 7:** Summary of centrality measures and top-ranked concepts.

Centrality measure	Analytical dimension	Top-ranked concept
Degree centrality	Discursive prominence	Cooperation
Closeness centrality	Structural accessibility	Financing and governance
Betweenness centrality	Discursive mediation	Learning and collaboration

Overall, the findings suggest that expert discourse on development financing does not exclude ethical concerns, but tends to situate them in structurally secondary positions, limiting their capacity to substantively reconfigure priorities and decision-making frameworks.

Taken together, these results and findings suggest that discursive structures do not merely reflect thematic priorities, but actively participate in constructing and stabilizing specific regimes of meaning. From a Critical Discourse Studies perspective, the observed network configuration can be interpreted as a relational dispositif through which certain rationalities are normalized, while alternative perspectives are rendered less visible or less actionable within the field of development financing.

## 4 Discussion

These findings are consistent with previous empirical studies that have shown how development financing discourse tends to be structured through technocratic and depoliticized frameworks (Taggart and Power, 2024; Tan, 2022). At the same time, the present study extends this literature by demonstrating that such dynamics are not only observable at the level of institutional narratives, but are also embedded in the relational structure of expert discourse. By combining discourse analysis with network-based approaches, the findings provide a novel empirical perspective on how semantic hierarchies and discursive centralities contribute to the stabilization of dominant development rationalities.

To strengthen the analytical articulation between the Ethical Diamond and the network structure, the findings can be interpreted through the relational positioning of the identified concepts. Highly central nodes, associated with notions such as governance, cooperation, or efficiency, can be understood as expressions of dominant rationalities and institutional configurations within the discursive field. In contrast, nodes located in peripheral positions, linked to rights, justice, or equity, correspond to dimensions more closely related to practices and experiences of dignity. Furthermore, nodes with high betweenness centrality operate as mediating elements that translate or reframe normative demands within technocratic frameworks. In this way, the network structure makes visible how the different dimensions of the Ethical Diamond are not equally represented, but hierarchically structured according to their position within the discourse.

In this sense, a key contribution of this study lies in showing that technocratic and governance-oriented discourses dominate even within an academic and civil society-oriented deliberative space, suggesting the extension of hegemonic development rationalities beyond formal institutional arenas.

The findings of this study indicate that development financing does not operate as a purely technical or neutral mechanism, but rather as a relational field structured by discourses, power positions, and ethical hierarchies. The combination of qualitative discourse analysis and social network analysis makes it possible to empirically demonstrate how certain narratives acquire centrality not only because of their thematic content, but also because of the structural position of the actors and concepts that produce and mediate them.

### 4.1 Discursive centrality and the production of hegemony

The structure of the discursive network reveals an uneven distribution of discursive power, in which a limited number of actors and conceptual frameworks concentrate central positions of emission and intermediation. This centrality is not merely descriptive; it can be understood as a form of relational power in the sense proposed by Freeman (Freeman, 1978), enabling certain nodes to influence which narratives are perceived as legitimate, reasonable, or viable within the field of development financing.

From a critical discourse perspective, this pattern is consistent with Fairclough’s argument that institutional discourses do not function as neutral arenas of exchange, but as mechanisms of hegemonic production through which specific interpretative frameworks become naturalized and appear as technical consensus (Fairclough, 1993, 2013). In this case, the structural prominence of narratives associated with cooperation, governance, and financial efficiency contributes to stabilizing an expert language that delimits what is considered fundable and shapes the priorities of development policy.

The findings also resonate with van Dijk’s analysis of discursive elites (van Dijk, 1993), as the network configuration suggests that certain actors and conceptual clusters possess greater capacity to influence the collective cognitive frames through which development and its financing are understood. The presence of peripheral actors and critical discourses within the network does not automatically translate into equivalent influence. This challenges institutional narratives that equate formal inclusion with effective participation. The observed hierarchical architecture indicates that plurality of voices coexists with structural asymmetries in meaning-making capacity, reproducing power dynamics characteristic of contemporary global governance (Scholte, 2020).

Within this configuration, discursive centrality appears to function as a mechanism through which normative frameworks compatible with dominant logics of international financing are consolidated, while alternative perspectives that question underlying power relations tend to occupy structurally peripheral positions with reduced capacity for circulation and translation into concrete decisions. This supports an interpretation of development financing as a deeply political field, in which control over discourse constitutes a key resource of governance.

The findings also suggest the possibility of processes of discursive incorporation, whereby critical vocabularies—such as participation, sustainability, or social justice—are integrated into dominant frameworks without substantially transforming the structural power relations that shape development financing. This incorporation does not eliminate critical discourse; rather, it may translate it into governance-compatible language, potentially limiting its transformative scope.

### 4.2 Ethics, instrumentalization, and normative imbalances

From a critical paradigm and drawing on the dimensions of the ethical diamond of human rights (Herrera Flores, 2008), the findings suggest the presence of persistent normative imbalances in the structure of expert discourse on development financing. Dimensions more closely aligned with dominant institutional logics—such as efficiency, organizational viability, impact measurement, and accountability—occupy structurally central positions within the discursive network. By contrast, those associated with human dignity, social justice, redistribution, and collective empowerment appear more fragmented and structurally displaced toward peripheral zones of the network.

This pattern does not indicate an absence of ethical references. Rather, it points toward a possible instrumental reconfiguration of ethics, consistent with what Herrera Flores describes as a progressive decoupling between rights, social conflict, and the material conditions of dignity. Within the analyzed discourse, ethics appears to function primarily as a language of legitimation compatible with technical financing frameworks, rather than as a space of critical deliberation oriented toward questioning power relations, structural asymmetries, or historically rooted processes of inequality. This interpretation is consistent with the structural positioning identified through centrality measures, which show weaker relational integration of explicitly justice-oriented concepts.

From a Foucauldian perspective, this dynamic can be interpreted as a process of discursive normalization, whereby certain ethical values become embedded within institutionalized regimes of truth that delimit what can be said and thought in the field of development (Foucault, 1972). Ethics does not disappear, but becomes inscribed within a framework privileging governability, management, and systemic stability, thereby potentially reducing its critical and transformative scope. In terms of Critical Discourse Analysis, this process contributes to the naturalization of a particular normative order, presenting it as technical, inevitable, or broadly consensual (Fairclough, 1993).

This ethical displacement is consistent with critiques from critical development studies, which argue that dominant discourses tend to depoliticize notions such as justice, equity, or sustainability by subordinating them to criteria of institutional effectiveness and performance (Escobar, 1998; Eyben, 2012). In the semantic network analyzed here, references to rights and social justice persist, but they occupy structurally weaker positions, with comparatively limited capacity to reconfigure the central frameworks of development financing.

Taken together, the findings suggest that development financing may be articulated around a functionalized ethical register—one oriented more toward stabilizing existing arrangements than toward directly confronting distributive tensions and social conflicts inherent in development processes. From critical development perspectives, this configuration can be interpreted as pointing toward a potential erosion of the link between ethics and structural transformation, thereby reopening the normative debate on development financing as a field of political contestation rather than solely a matter of technical management or institutional design.

### 4.3 Discourse, technocracy, and the depoliticization of development

The findings of this study point to a clear predominance of technocratic discursive frameworks that construct development financing primarily as a matter of management, innovation, and resource optimization. Such framing tends to displace the political and conflictive dimensions of development, presenting deeply normative decisions as technical, neutral, or inevitable responses. As critical development theories have long argued, this displacement does not eliminate conflict, but rather discursively deactivates it by relocating it outside the realm of legitimate debate (Escobar, 1998; Sachs, 1992).

From a Foucauldian perspective, the discursive network can be interpreted as a dispositif of normalization, within which certain vocabularies consolidate as regimes of truth that delimit what can be said, thought, and ultimately financed in the field of development (Foucault, 1972). The structural centrality of these concepts is not merely descriptive; it reflects their capacity to organize the discursive field, stabilize consensus, and narrow the space available for dissent and substantive alternatives.

Critical Discourse Analysis allows this dynamic to be understood as a process of structural depoliticization, in the sense outlined by Fairclough (1993): dominant discourses transform complex social problems into technical issues, shifting debate away from justice, power, and redistribution toward expert management. In the network examined here, this logic is reflected in the strong centrality of concepts associated with institutional coordination and governance, alongside the structural relegation of narratives that question the foundational assumptions of prevailing financing models.

The observed coherence between the structural position of actors and the types of discourse they articulate further reinforces this interpretation. More critical narratives—linked to inequality, rights, or social justice—tend to occupy peripheral positions within the network, displaying limited capacity for circulation and translation into operational decision-making frameworks. This configuration suggests that the network structure not only conditions who speaks, but also which discourses become audible, legitimate, and actionable, and which remain confined to marginal spaces of reflection or critique.

As Eyben (2012) argues, technocratic languages of cooperation and financing are not politically neutral; they operate as mechanisms of discursive closure that constrain the articulation of situated knowledges and alternative rationalities. Similarly, Scholte (2020) notes that the construction of “the global” as a technical and autonomous sphere contributes to the depoliticization of decisions that have profound material consequences for specific territories and communities. The findings presented here provide empirical support for this claim, showing how depoliticization operates not only through policy documents, but also through relational discursive structures that hierarchize concepts and marginalize conflict.

Taken together, the analyzed discursive network suggests that development financing is framed as a domain of technical consensus, while functioning in practice as a deeply political arena structured by power relations, exclusion, and normative contestation. The observed depoliticization does not eliminate ethics from the discourse, but shapes the kinds of ethical claims that can circulate, privileging formulations compatible with technocratic governance and potentially constraining the transformative scope of critical and ecosocial approaches.

### 4.4 Implications and limitations of the study

The findings of this study carry relevant implications for the critical analysis of development financing, both at theoretical and methodological levels.

First, they highlight the need to rethink dominant participatory approaches, which often assess inclusion in terms of formal actor presence while overlooking the effective distribution of discursive power. As critical discourse and global governance theories suggest, participation does not in itself guarantee influence if the rules of discursive engagement, legitimate vocabularies, and intermediary nodes remain concentrated in dominant positions (Fairclough, 1993; van Dijk, 1993; Scholte, 2020). The network analysis presented here provides empirical evidence of how such asymmetries can persist even within deliberative spaces framed as inclusive.

Second, the study underscores the analytical value of critical human rights and critical development perspectives as normative lenses for identifying processes of ethical hierarchization within complex institutional settings. Rather than functioning as a classificatory or evaluative instrument, the Ethical Diamond enables the identification of how certain dimensions—aligned with efficiency, governance, and accountability logics—acquire structural centrality, while others linked to dignity, social justice, redistribution, and transformative empowerment appear fragmented or displaced. In this sense, the findings confirm that ethics is not absent from development financing discourse, but is frequently reconfigured instrumentally as a language of legitimation, consistent with critiques advanced in critical development and human rights scholarship (Herrera Flores, 2008; Escobar, 1998; Eyben, 2012).

From a methodological standpoint, the combination of Critical Discourse Analysis and semantic network analysis demonstrates its capacity to empirically operationalize relations of discursive power, moving beyond purely textual or descriptive interpretations. The use of centrality measures makes it possible to identify not only which concepts are most visible, but which narratives function as semantic infrastructures within expert discourse, and which remain confined to peripheral positions with limited capacity for institutional translation (Freeman, 1978). This integrative approach contributes to narrowing the gap between critical discourse theory and empirical analysis of global governance processes.

At the same time, several limitations should be acknowledged. The situated nature of the study, based on a specific corpus of actors and discourses linked to a particular academic event, calls for caution in generalizing the findings. Moreover, the analysis focuses on discursive configurations and does not directly examine how these relational structures translate into concrete financial decisions or material impacts on territories. Future research could extend this approach comparatively or longitudinally, and integrate participatory, ethnographic, or action-research methodologies to explore how these discursive networks are experienced, appropriated, or contested by social organizations and affected communities.

Overall, this study contributes to a deeper understanding of development financing as a field of ethical and political contestation rather than a purely technical problem of resource allocation. The findings indicate that financial decisions not only distribute funds, but also configure meanings, priorities, and horizons of social transformation, delimiting which development models become thinkable, fundable, and legitimate. From this perspective, critically analyzing the discursive structures underpinning development financing becomes central to opening spaces for democratization, justice, and sustainability within global governance.

## 5 Conclusion

This study demonstrates that development financing does not operate as a neutral or purely technical mechanism, but as a discursive field structured by power relations, ethical hierarchies, and processes of depoliticization. By integrating critical discourse analysis with semantic network analysis, the research shows that discursive centrality depends less on the normative content of particular concepts than on their structural position within the relational architecture of expert debate.

The findings reveal a hierarchical discursive configuration in which concepts associated with cooperation, governance, efficiency, and sustainability occupy central articulating positions. These narratives function as semantic infrastructures that delimit what is thinkable, legitimate, and ultimately financeable within the field of development. In contrast, discourses linked to human rights, social justice, and inequality are positioned peripherally, with comparatively limited structural capacity to influence the overall configuration of meaning.

The incorporation of centrality metrics and network analysis makes it possible to visualize how such hierarchies are structurally stabilized beyond mere textual frequency. Discursive power is not only a matter of visibility, but of relational positioning and mediation capacity within the network.

From the perspective of critical human rights theory, the findings point to persistent normative imbalances that privilege dimensions aligned with dominant institutional logics—such as efficiency, organizational viability, impact measurement, and accountability—while marginalizing those oriented toward redistribution, dignity, and structural transformation. Ethical references remain present within expert discourse, yet they tend to be reconfigured instrumentally as languages of legitimacy, partially detached from social conflict and structural power asymmetries.

At the same time, the predominance of technocratic frames reinforces the depoliticization of development, presenting deeply normative decisions as technical and inevitable solutions. This configuration constrains the circulation of critical and ecosocial rationalities and consolidates governance models that reduce space for dissent and substantive alternatives.

The main contribution of this article lies in demonstrating the analytical value of combining relational network analysis with critical ethical frameworks to examine development financing as a site of political and normative contestation. Advancing toward more just and democratic financing models therefore requires not only institutional or financial reform, but also a reconfiguration of the discursive structures through which development priorities are defined and legitimized in contemporary global governance.

## Data Availability

The raw data supporting the conclusions of this article will be made available by the authors, without undue reservation.
