# Supplementary material for: Discursive structures of global development governance: a mixed-methods network analysis of an academic deliberative space in the context of FfD4
Source: Front Sociol. 2026 May 29;11:1813491. doi: 10.3389/fsoc.2026.1813491 (PMC13281702; doi:10.3389/fsoc.2026.1813491)
Supplement: Supplementary file 1 [file Data_Sheet_1.pdf]

Appendix A. Bilingual Codebook of Analytical Categories (Spanish–English)

Nº	SPANISH	ENGLISH
**1**	Agenda 2030	2030 Agenda
**2**	Agradecimiento	Acknowledgment
**3**	Análisis crítico	Critical analysis
**4**	Aprendizaje y colaboración	Learning and collaboration
**5**	Aprendizaje y colaboración: Cocreación	Learning and collaboration: Co-creation
**6**	Aprendizaje y colaboración: Construcción de confianza	Learning and collaboration: Trust-building
**7**	Aprendizaje y colaboración: Diálogo interdisciplinario	Learning and collaboration: Interdisciplinary dialogue
**8**	Aprendizaje y colaboración: Diversidad de datos	Learning and collaboration: Data diversity
**9**	Aprendizaje y colaboración: Diversidad de experiencias	Learning and collaboration: Diversity of experiences
**10**	Aprendizaje y colaboración: Efectividad	Learning and collaboration: Effectiveness
**11**	Aprendizaje y colaboración: Experiencia local	Learning and collaboration: Local experience
**12**	Aprendizaje y colaboración: Identificación de riesgos	Learning and collaboration: Risk identification
**13**	Aprendizaje y colaboración: Importancia de las competencias	Learning and collaboration: Importance of skills
**14**	Aprendizaje y colaboración: Interés en redes	Learning and collaboration: Interest in networks
**15**	Aprendizaje y colaboración: Mejoramiento de la comunicación	Learning and collaboration: Communication improvement
**16**	Aprendizaje y colaboración: Modelización	Learning and collaboration: Modeling
**17**	Aprendizaje y colaboración: Mujeres en el trabajo	Learning and collaboration: Women in the workplace
**18**	Aprendizaje y colaboración: Necesidad de profundizar	Learning and collaboration: Need for further exploration
**19**	Aprendizaje y colaboración: Oportunidades de red	Learning and collaboration: Networking opportunities
**20**	Aprendizaje y colaboración: Planificación de actividades	Learning and collaboration: Activity planning
**21**	Aprendizaje y colaboración: Presentación de panel	Learning and collaboration: Panel presentation
**22**	Aprendizaje y colaboración: Prevención de problemas escolares	Learning and collaboration: Prevention of school problems
**23**	Aprendizaje y colaboración: Priorizar soluciones	Learning and collaboration: Prioritizing solutions
**24**	Aprendizaje y colaboración: Redes neuronales	Learning and collaboration: Neural networks
**25**	Autonomía	Autonomy
**26**	Cambio	Change
**27**	Cambio climático	Climate change
**28**	Cambio social	Social change
**29**	Cambio y desarrollo	Change and development
**30**	Cambio y desarrollo: Desafíos actuales	Change and development: Current challenges
**31**	Cambio y desarrollo: Proceso de superación	Change and development: Overcoming process
**32**	Cambio y desarrollo: Proceso de transformación	Change and development: Transformation process
**33**	Cambio y desarrollo: Tecnología y desarrollo	Change and development: Technology and development
**34**	Cambio y desarrollo: Transición de tema	Change and development: Topic transition
**35**	Colaboración	Collaboration
**36**	Complejidad	Complexity
**37**	Compromiso social	Social commitment
**38**	Comunicación	Communication

Appendix A. Bilingual Codebook of Analytical Categories (Spanish–English)

**39**	Cooperación	Cooperation
**40**	Cooperación internacional	International cooperation
**41**	Crítica social	Social critique
**42**	Crítica y reflexión	Critique and reflection
**43**	Crítica y reflexión: Crítica a modelos existentes	Critique and reflection: Critique of existing models
**44**	Crítica y reflexión: Evaluación de situaciones	Critique and reflection: Situation assessment
**45**	Crítica y reflexión: Literatura académica	Critique and reflection: Academic literature
**46**	Crítica y reflexión: Referencias sociológicas	Critique and reflection: Sociological references
**47**	Crítica y reflexión: Reflexión sobre la economía	Critique and reflection: Reflection on the economy
**48**	Crítica y reflexión: Teorías feministas	Critique and reflection: Feminist theories
**49**	Crítica y reflexión: Valoración de la otra persona	Critique and reflection: Valuing others
**50**	Derechos humanos	Human rights
**51**	Derechos y equidad	Rights and equity
**52**	Derechos y equidad: Equidad económica	Rights and equity: Economic equity
**53**	Derechos y equidad: Lucha por la propiedad	Rights and equity: Struggle for property
**54**	Derechos y equidad: Necesidad de equidad de género	Rights and equity: Need for gender equity
**55**	Derechos y equidad: Tensiones éticas	Rights and equity: Ethical tensions
**56**	Derechos y equidad: Violencia y derechos humanos	Rights and equity: Violence and human rights
**57**	Desarrollo comunitario	Community development
**58**	Desarrollo sostenible	Sustainable development
**59**	Desarrollo territorial	Territorial development
**60**	Descentralización	Decentralization
**61**	Desigualdad	Inequality
**62**	Desigualdad social	Social inequality
**63**	Desigualdad social (2)	Social inequality (2)
**64**	Desigualdad social: Desigualdades de poder	Social inequality: Power inequalities
**65**	Desigualdad social: Dificultades democráticas	Social inequality: Democratic difficulties
**66**	Desigualdad social: Impacto de la exclusión	Social inequality: Impact of exclusion
**67**	Desigualdad social: Limitaciones en el desarrollo	Social inequality: Development constraints
**68**	Desigualdad social: No hay códigos aplicables	Social inequality: No applicable codes
**69**	Diálogo	Dialogue
**70**	Economía social	Social economy
**71**	Empoderamiento	Empowerment
**72**	Empoderamiento femenino	Women’s empowerment
**73**	Falta de información	Lack of information
**74**	Financiación	Financing
**75**	Financiamiento	Funding
**76**	Financiamiento y gobernanza	Financing and governance
**77**	Financiamiento y gobernanza: Complejidad financiera	Financing and governance: Financial complexity
**78**	Financiamiento y gobernanza: Democratización	Financing and governance: Democratization
**79**	Financiamiento y gobernanza: Derivación a recursos	Financing and governance: Resource allocation
**80**	Financiamiento y gobernanza: Diálogo entre grupos de interés	Financing and governance: Stakeholder dialogue

Appendix A. Bilingual Codebook of Analytical Categories (Spanish–English)

**81**	Financiamiento y gobernanza: Economía sólida	Financing and governance: Strong economy
**82**	Financiamiento y gobernanza: Finanzas sociales	Financing and governance: Social finance
**83**	Financiamiento y gobernanza: Foros internacionales	Financing and governance: International forums
**84**	Financiamiento y gobernanza: Generación de recursos	Financing and governance: Resource generation
**85**	Financiamiento y gobernanza: Gestión de residuos	Financing and governance: Waste management
**86**	Financiamiento y gobernanza: Gobernanza democrática	Financing and governance: Democratic governance
**87**	Financiamiento y gobernanza: Intervención extranjera	Financing and governance: Foreign intervention
**88**	Financiamiento y gobernanza: Mecanismos de compra	Financing and governance: Procurement mechanisms
**89**	Financiamiento y gobernanza: Objetivos globales	Financing and governance: Global objectives
**90**	Financiamiento y gobernanza: Procesos administrativos	Financing and governance: Administrative processes
**91**	Financiamiento y gobernanza: Reconocimiento institucional	Financing and governance: Institutional recognition
**92**	Frustración	Frustration
**93**	Gobernanza	Governance
**94**	Impacto social	Social impact
**95**	Inclusión	Inclusion
**96**	Innovación	Innovation
**97**	Interacción social	Social interaction
**98**	Interdisciplinariedad	Interdisciplinarity
**99**	Intervención social	Social intervention
**100**	Investigación	Research
**101**	Justicia social	Social justice
**102**	Liderazgo	Leadership
**103**	Necesito más contexto	Need more context
**104**	Participación	Participation
**105**	Participación activa	Active participation
**106**	Participación ciudadana	Citizen participation
**107**	Participación comunitaria	Community participation
**108**	Planificación	Planning
**109**	Pobreza	Poverty
**110**	Política pública	Public policy
**111**	Políticas públicas	Public policies
**112**	Reconocimiento	Recognition
**113**	Reflexión	Reflection
**114**	Reflexión crítica	Critical reflection
**115**	Responsabilidad	Responsibility
**116**	Solidaridad	Solidarity
**117**	Sostenibilidad	Sustainability
**118**	Toma de decisiones	Decision-making
**119**	Trabajo en equipo	Teamwork
**120**	Transformación social	Social transformation
**121**	Transparencia	Transparency
